# The complete chloroplast genome of *Berberis weiningensis* (Berberidaceae), an endemic and traditional Chinese medicinal herb

**DOI:** 10.1080/23802359.2021.1901625

**Published:** 2021-03-24

**Authors:** Tu Feng, Qun-Ying Xiao, Wang-Jun Li, Chun Zhang, Yang He, Gang Fan

**Affiliations:** a School of Ecological Engineering, Guizhou University of Engineering Science, Bijie, PR China; b School of Medical Technology, Chengdu University of Traditional Chinese Medicine, Chengdu, PR China; c School of Ethnic Medicine, Chengdu University of Traditional Chinese Medicine, Chengdu, PR China

**Keywords:** Chloroplast genome, *Berberis weiningensis*, Berberidaceae

## Abstract

*Berberis weiningensis* is a frequently-used traditional Chinese medicinal herb that included various active alkaloids. In this study, we assembled the complete chloroplast (cp) genome of *B. weiningensis*. The complete cp genome of *B. weiningensis* is 166,275 bp in length, and has a typical structure with large single-copy (LSC 73,624 bp) and small single-copy (SSC 18,608 bp) regions separated by a pair of inverted repeats (IRs 37,019 bp) of large size. The *B. weiningensis* cp genome contains 147 genes, of which 101 protein-coding genes, 8 rRNA genes, and 38 tRNA genes. The phylogenetic analysis revealed that *Berberis* species closely clustered with *Mahonia* species, which obviously support that Mahonia and Berberis are not monophyletic.

*Berberis* Linnaeus (1753) was a shrubs, evergreen, or deciduous taxonomical genus in Berberidaceae. There were about 500 species distributed in north temperate regions, a few in the S Hemisphere; 215 species (197 endemic, one introduced) in China and many species of the genus were grown as ornamental shrubs and used for medicinal purposes (Ying et al. 2011).

In China, several *Berberis* species have medicinal properties because of the presence of various active alkaloids. The alkaloids in *Berberis* herbs included berberine, magnoflorine, and palmatine, which were recognized as the main active constituents. And the biological activities of alkaloids in *Berberis* herbs were consistent with the pharmacological effects (Xu et al. 2020).

*Berberis weiningensis* were evergreen shrubs, and have been used for the treatment of diarrhea, urinary frequency, diabetes, trachoma, gastritis, and nephritis for centuries in traditional medicinal system. *B. weiningensis* was an endemic plant and mainly distributed in weedy places on mountain summits which 2100–2500 m in Guizhou province, southern China (Ying et al. 2011; Feng et al. 2018).

In this study, we made the first report of a complete plastome for *B. weiningensis*. The annotated chloroplast genome sequence has been deposited into GenBank with the accession number MW018363.

The mature leaves of *B. weiningensis* were collected from Daduzi mountain, Weining county, Bijie City, Guizhou Province, China (104°10′1.42″N and 26°49′37.8″E) and voucher specimens (20170625022) were deposited at BJ (Bijie University Herbarium, Bijie City, Guizhou Province, China). Morphological characters were measured using MAML version 1.0 (Altinordu et al. 2016) and contrasted in the National Specimen Information Infrastructure, specimen platform of China, teaching specimens sub-platform (Web, http://mnh.scu.edu.cn/, 2005DKA21403-JK). Total genomic DNA was extracted from the silica-dried leaves using the TIANGEN plant genomic DNA extraction kit, following the manufacturer’s instructions. The genomic paired-end (PE150) sequencing was performed on an Illumina Hiseq 2000 instrument (Illumina, San Diego, CA). The complete cp genome was assembled using SOAPdenovo2 (Luo et al. 2012) and the resulting contigs were linked based on overlapping regions after being aligned to *Berberis koreana* (NC 030063) using Geneious Prime version 2020.0.3. Annotation was performed via Geneious Prime 2020.0.3, coupled with manual check and adjustment.

The complete plastome of *B. weiningensis* is 166,275 bp in length, including two single-copy regions (LSC: 73,624 bp and SSC: 18,608 bp) and two inverted repeat regions (IRs: 37,019 bp). The complete chloroplast genome sequence of the *B. weiningensis* contains a pair of especially large IRs that was also found in *B. koreana* (NC 030063). The whole GC content of the total length, LSC, SSC, and IR regions is 38.0%, 36.5%, 32.6%, and 40.9%, respectively. It contained 147 genes, including 101 protein-coding genes, 8 rRNA genes, and 38 tRNA genes were annotated. Thirty-two genes are duplicated in the IR regions, which is congruent with *B. fortunei* and *Mahonia bealei* (Ma et al. 2013).

The phylogeny was reconstructed based on 38 Berberidaceae species, using maximum likelihood (ML). The sequences were aligned using MAFFT version 7 (Katoh et al. 2017), and RAxML version 8.2.10 (Stamatakis 2014) were used to construct a maximum likelihood tree. The phylogenetic analysis revealed that *Berberis* species closely clustered with *Mahonia* species (Figure 1), but the *Berberis* and *Mahonia* are strongly supported as monophyletic based on nuclear marker (ITS) and five plastid regions(Chen et al. 2020). The results indicated that the phylogenetic researches need redefinition based on different markers between *Berberis* and *Mahonia* genera (Xiao et al. 2020).

**Figure 1.**
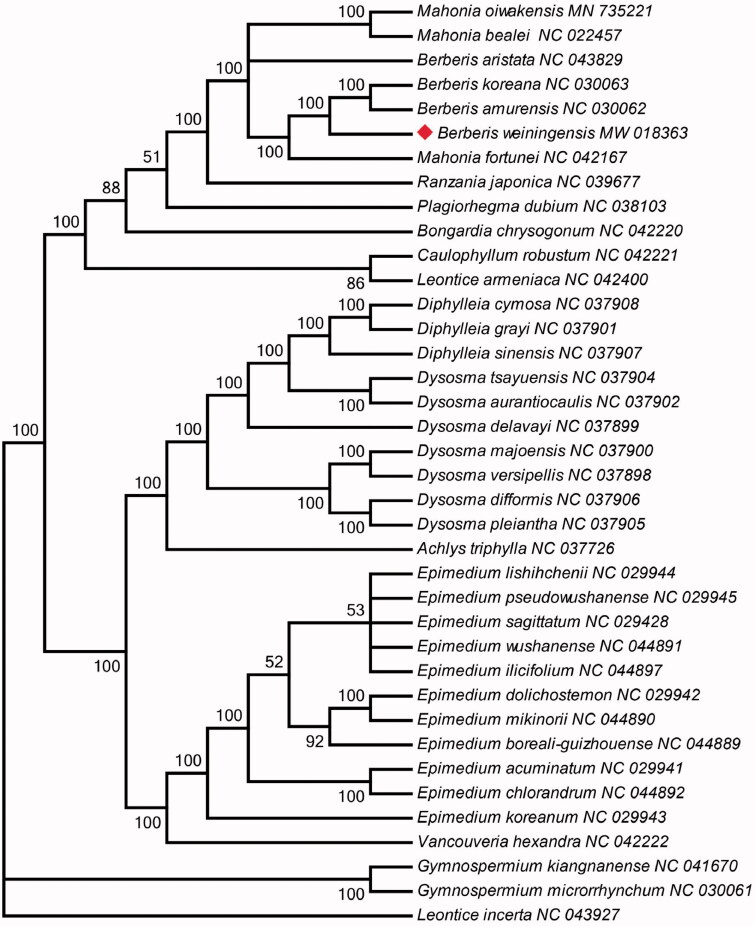
The best ML phylogeny recovered from 38 complete plastome sequences by RAxML. Numbers on the nodes are bootstrap values from 1000 replicates and ML bootstrap values <50% were not shown. The symbol ♦ means *Berberis weiningensis* in this study.

Berberidaceae was divided into several groups based on chromosome base number, which was consistent with previous molecular phylogenetic studies on Berberidaceae (Kim, Kim, Kim, et al. 2004; Kim, Kim, Landrum 2004). *Gymnospermium* species formed into a high bootstrap values (100%) branch which had chromosome base number with *x* = 8. The five genera *Diphylleia*, *Dysosma*, *Achlys*, *Epimedium*, and *Vancouveria* formed into a large branch, which had the high bootstrap values (100%) and chromosome base number with *x* = 6. The another branch included *Mahonia*, *Berberis*, *Ranzania*, *Plagiorhegma*, *Bongardia, Leontice*, and *Caulophyllum*. In this branch, the high bootstrap values (100%) sub-branch included *Mahonia*, *Berberis*, and *Ranzania* which had chromosome base number with *x* = 7. The *Leontice* and *Caulophyllum* were clustered into a branch which had chromosome base number with *x* = 8 (Huang et al. 2019).

The possible reason for the clustering of *Mahonia* and *Berberis* maybe that have the approximate characteristic of morphology and alkaloids. Some taxonomic research treated several compound leaved *Mahonia* species in the simple-leaved *Berberis* group. In this study, the phylogenetic analysis of the complete chloroplast genome implied that *Mahonia* and *Berberis group* is non monophyly although *Mahonia* was recognized as a separate genus in *Flora of China*. It indicated that the hypothesis that woodiness in *Berberis* and *Mahonia* originated from a herbaceous ancestor and this is a difficult distribution to explain (Kim, Kim, Kim, et al. 2004; Kim, Kim, Landrum 2004).

## Data Availability

The genome sequence data that support the findings of this study are openly available in GenBank of NCBI at (https://www.ncbi.nlm.nih.gov/) under the accession no. MW018363. The associated BioProject, SRA, and Bio-Sample numbers are PRJNA701777, SRX10128792, and SAMN17910888, respectively.
